# Intravenous thrombolysis versus antiplatelet therapy in minor stroke patients with large vessel occlusion

**DOI:** 10.1111/cns.14124

**Published:** 2023-03-07

**Authors:** Chunmiao Duan, Yunyun Xiong, Hongqiu Gu, Shang Wang, Kai‐Xuan Yang, Manjun Hao, Xueyan Feng, Xingquan Zhao, Xia Meng, Yongjun Wang

**Affiliations:** ^1^ Vascular Neurology, Department of Neurology Beijing Tiantan Hospital, Capital Medical University Beijing China; ^2^ China National Clinical Research Center for Neurological Diseases Beijing China; ^3^ Department of Neurology Beijing Daxing Hospital, Capital Medical University Beijing China; ^4^ Chinese Institute for Brain Research Beijing China; ^5^ Neurocardiology Center, Department of Neurology Beijing Tiantan Hospital, Capital Medical University Beijing China; ^6^ Center for Stroke Beijing Institute for Brain Disorders Beijing China

**Keywords:** aspirin, large vessel occlusion, stroke, thrombolysis

## Abstract

**Aim:**

Our study aimed to explore the effectiveness and safety of intravenous t‐PA compared with dual antiplatelet therapy (DAPT) and aspirin alone for minor stroke with National Institutes of Health Stroke Scale (NIHSS) score ≤5 and large vessel occlusion (LVO).

**Methods:**

Patients with minor stroke harboring LVO within 4.5‐h time window were included from the Third China National Stroke Registry (CNSR‐III) between August 2015 and March 2018 in China. Clinical outcomes including modified Rankin scale (mRS) score, recurrent stroke, and all‐cause mortality at 90 days and 36‐h symptomatic intracerebral hemorrhage (sICH) were collected. Multivariable logistic regression models and propensity score matching analyses were used to determine the association between treatment groups and clinical outcomes.

**Results:**

A total of 1401 minor stroke patients with LVO were included. Overall 251 patients (17.9%) received intravenous t‐PA, 722 patients (51.5%) received DAPT, and 428 patients (30.5%) received aspirin alone. The intravenous t‐PA was associated with greater proportions of mRS 0–1 (aspirin versus t‐PA: adjusted odds ratio [aOR], 0.50; 95% confidence interval [CI], 0.32 to 0.80; *p* = 0.004; DAPT versus t‐PA: aOR, 0.76; 95% CI, 0.49 to 1.19; *p* = 0.23). Using propensity score matching analyses, the results were similar. There was no difference in 90‐day recurrent stroke among the groups. The rates of all‐cause mortality in intravenous t‐PA, DAPT, and aspirin groups were 0%, 0.55%, 2.34%, respectively. No patient developed sICH within 36 h of intravenous t‐PA.

**Conclusion:**

In patients with minor stroke harboring LVO within 4.5‐h time window, intravenous t‐PA was associated with higher odds for the excellent functional outcome, as compared with the aspirin alone. Further randomized controlled trials are warranted.

## Graphical abstract

Of 1401 patients with NIHSS ≤5 harboring LVO admitted within 4.5‐h time window, intravenous t‐PA was better than aspirin alone, but similar with DAPT for achieving 90‐day excellent functional outcome (defined as modified Rankin Scale [mRS] of 0–1) by multivariable logistic regression analysis.

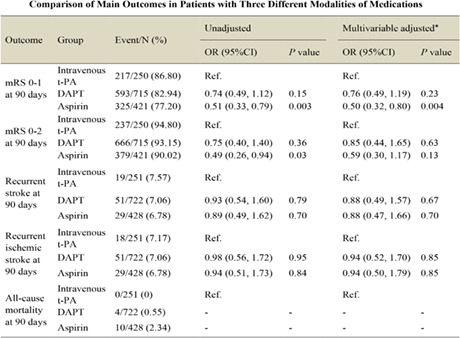

## 1 INTRODUCTION

About 30% of patients with minor stroke have a 90‐day functional disability.^1^ Even though the Clopidogrel in High‐Risk Patients with Acute Nondisabling Cerebrovascular Events (CHANCE)^2^ and the Platelet‐Oriented Inhibition in New TIA and Minor Ischemic Stroke (POINT)^3^ trials have shown early clopidogrel plus aspirin could reduce the risk of 90‐day recurrent stoke for minor stroke with NIHSS ≤3 and high‐risk TIA with ABCD2 ≥ 4 by 32% and 27%, respectively, minor stroke are still at high risk of short‐ and long‐term poor functional outcomes and recurrent stroke.^4^, ^5^

Patients with acute ischemic stroke and large vessel occlusion (LVO) might present with low NIHSS scores, partly due to good collateral status.^6^ However, these patients might be at high risk of deterioration.^7^, ^8^ The CT And MRI in the Triage of TIA and minor cerebrovascular events to identify High‐risk patients (CATCH) study^9^ showed that the presence of symptomatic intracranial or extracranial vessel occlusion or stenosis ≥50% was the main predictor for deterioration and once aggravated, even if given a secondary reperfusion therapy (intravenous thrombolysis and/or endovascular treatment), the 90‐day functional outcome of these patients was not better than primary reperfusion therapy.^10^, ^11^

Up to now, there is limited evidence of thrombolysis for acute minor stroke with LVO. The Potential of rt‐PA for Ischemic Strokes with Mild Symptoms (PRISMS) trial was a phase 3, randomized, double‐blinded trial which compared intravenous t‐PA to aspirin in non‐disabling minor ischemic stroke.^12^ Nevertheless, the trial was terminated prematurely due to slow enrollment and did not include LVO. The 2019 updated guidelines for the early management of acute ischemic stroke from the American Heart Association/American Stroke Association^13^ and the 2021 European guidelines for intravenous thrombolysis in acute ischemic stroke^14^ could not provide evidence‐based recommendations. We aimed to compare the clinical effectiveness and safety of minor ischemic stroke with LVO receiving intravenous t‐PA, dual antiplatelet treatment (DAPT), and aspirin alone. We hypothesized that intravenous t‐PA might be associated with better 90‐day functional outcomes than DAPT or aspirin alone in minor ischemic stroke with LVO.

## 2 METHODS

The CNSR‐III, designed to establish the etiology, imaging, and biology markers for clarifying the pathogenesis and prognostic factors of ischemic stroke, was a nationwide prospective registry for patients presented to hospitals with acute ischemic stroke (AIS) and transient ischemic attack (TIA) between August 2015 and March 2018 in China. All patients with age older than 18 years fulfilling the criteria of diagnosis of AIS or TIA within 7 days were consecutively enrolled. Trained research coordinators utilized an electronic data capture system to collect data and paper‐based case report forms were recorded if an electronic data capture system was not available. Independent contract research organization monitored the data independently throughout the study. All data were de‐identified before data analysis. The detailed design, rationale, and basic description of the CNSR‐III have been published previously.^15^ The study was approved by the Institutional Review Board and informed consent from patient or legally authorized representative was required.

### 2.1 Study population

A total of 15,166 patients were included from 201 hospitals of 22 provinces and four municipalities in China in CNSR‐III. We derived the patients fulfilling the following inclusion criteria: (1) minor ischemic stroke: defined as ischemic stroke with NIHSS score of 0 to 5 at admission, regardless of specific symptoms; (2) LVO: an occlusion of internal carotid arteries (ICA), middle cerebral arteries (MCA), anterior cerebral arteries (ACA), posterior cerebral arteries (PCA), vertebral arteries (VA) or basilar artery (BA) identified on magnetic resonance angiography (MRA), computed tomography angiography (CTA), or digital subtraction angiography (DSA) at admission as read and adjudicated centrally; (3) time window: defined as presenting with stroke symptoms within 4.5 h of symptom onset or within 4.5 h of awakening after the point when last seen normal; (4) treated with intravenous t‐PA, DAPT, or aspirin alone within 24 h. Aspirin (a dose of 100 mg per day) was given within 24 h after symptom onset. DAPT was defined as aspirin plus clopidogrel (aspirin at a dose of 100 mg per day, plus clopidogrel at an initial dose of 75 mg or 300 mg, followed by 75 mg per day) within 24 h after symptom onset. Intravenous t‐PA (0.9 mg/kg to a maximum dose of 90 mg, with 10% as initial bolus and the remainder over 1 h intravenous infusion) was administered within 4.5‐h time window. The exclusion criteria were (1) admission diagnosis of TIA and (2) endovascular therapy including arterial thrombolysis and mechanical thrombectomy. We classified the included patients into disabling and non‐disabling stroke group in the further subgroup analysis. Disabling stroke was defined as complete hemianopsia (≥2 on the NIHSS Question #3), or severe aphasia (≥2 on NIHSS Question #9), or visual or sensory extinction (≥1 on NIHSS Question #11), or any weakness limiting sustained effort against gravity (≥2 on NIHSS Question #6 or #7) or any consciousness disorder (≥1 on NIHSS Question #1a).^16^

### 2.2 Data collection and definitions

Patients were divided into intravenous t‐PA, DAPT, and aspirin alone groups based on their firstly initiated treatment. We extracted the following variables: demographics (including age, sex, and weight), medical history (including current smoking, hypertension, diabetes mellitus, dyslipidemia, coronary heart disease/myocardial infarction, atrial fibrillation, prior TIA, prior stroke), prior mRS (range, 0 to 6, with lower scores indicating better functional status), medication history (including antiplatelet, anticoagulant, and lipid‐lowering agents), baseline random blood glucose, baseline systolic blood pressure, baseline diastolic blood pressure, stroke severity (measured by NIHSS, range, 0 to 42, with higher scores indicating severe stroke, including the score of subitems), onset to treatment time, etc. Etiology classification of ischemic stroke was based on an expanded version of the TOAST (Trial of Org 10,172 in Acute Stroke Treatment) classification.^17^

### 2.3 Assessments

The primary outcome was an excellent functional outcome which was defined as mRS score of 0–1 at 90 days. The secondary outcomes included favorable functional outcome (defined as mRS score of 0–2 at 90 days), recurrent stroke at 90 days, recurrent ischemic stroke at 90 days, and all‐cause mortality at 90 days. The safety outcomes included rates of (1) symptomatic intracerebral hemorrhage (sICH) within 36 h: defined by the European Cooperative Acute Stroke Study III (ECASS III) criteria, any apparently extravascular blood in the brain or within the cranium, and clinical deterioration with an increase in the NIHSS of at least 4 points or any intracerebral hemorrhage leading to death;^18^ (2) life‐threatening or serious systemic hemorrhage within 36 h: defined by the Global Utilization of Streptokinase and t‐PA for Occluded Coronary Arteries (GUSTO) criteria, bleeding resulting in substantial hemodynamic imbalance requiring treatment.^19^

### 2.4 Statistical analyses

The data were tested for normal distribution using the Kolmogorov–Smirnov test. Continuous variables were summarized as mean ± standard deviation (SD) if the distribution was normal or as median with interquartile range (IQR) otherwise, and differences were assessed using the ANOVA test whether normally distributed or non‐parametric test. Categorical variables were presented as frequencies with percentages, and the 𝝌^2^ test was used to compare the distributions between groups. Clinical outcomes (mRS 0–1, mRS 0–2, recurrent stroke, recurrent ischemic stroke and all‐cause mortality) were compared using a multivariable logistic regression after adjusting for confounding factors that were significant at the *p* < 0.1 level in the univariate analysis. To test the robustness of our results, subgroup analyses in disabling or not, NIHSS 0–2 and NIHSS 3–5 were further performed using the same statistical methods. Additional propensity score matching (PSM) analyses were performed to balance the baseline characteristics in the whole cohort, and symptomatic LVO, separately. And 1:1 matching was performed based on the nearest‐neighbor matching algorithm with a caliper width of 0.25. Two‐sided *p* < 0.05 was considered statistically significant. All analyses were performed using the SAS, version 9.4, software (SAS Institute, Cary, NC, USA).

## 3 RESULTS

### 3.1 Patient flowchart

**FIGURE 1:**
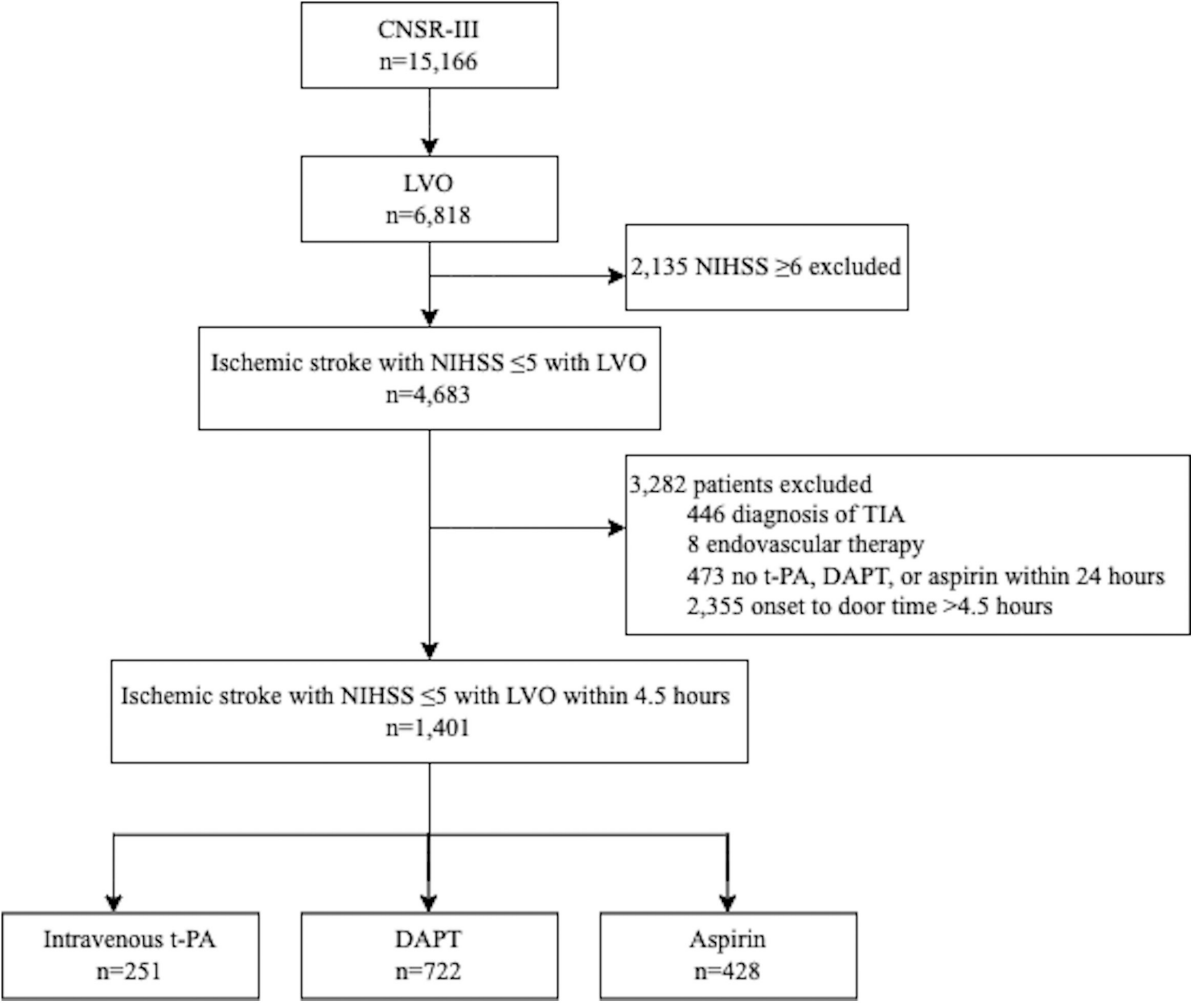
Study flowchart. CNSR‐III indicates the Third China National Stroke Registry; LVO, large vessel occlusion; NIHSS, National Institutes of Health Stroke Scale; TIA, transient ischemic attack; t‐PA, tissue‐type plasminogen activator; DAPT, dual antiplatelet treatment.

A total of 1401 patients with minor stroke (NIHSS ≤5) and LVO within 4.5‐h time window were included for subsequent analysis and in Figure 1 the flowchart depicts the reasons for patient exclusion, including no LVO on baseline vessel imaging (*n* = 8348), NHISS ≥6 (*n* = 2135), TIA (*n* = 446), endovascular therapy including arterial thrombolysis and mechanical thrombectomy (*n* = 8), no intravenous t‐PA, DAPT or aspirin within 24 h (*n* = 473), and onset to door time >4.5 h (*n* = 2355). Finally, 251 patients (17.9%) received intravenous t‐PA, 722 patients (51.5%) received DAPT, and 428 patients (30.5%) received aspirin alone.

### 3.2 Baseline characteristics

The baseline characteristics of the 1401 patients are described in Table 1. The medium onset to needle time was 2.9 h (interquartile range: 2.1–3.7). Patients treated with intravenous t‐PA among three groups were the youngest, had the highest proportion of current smoking, atrial fibrillation, and pre‐mRS score of 0–1, had the lowest proportion of diabetes mellitus, taking antiplatelet and lipid‐lowering agents, and had the highest baseline NIHSS. They are also more likely to arrive earlier at hospital and more likely to be treated in stroke unit. Of the studied cohort, LVO was diagnosed by MRA (86.9% [1218/1401]), CTA (12.6% [176/1401]), and DSA (0.5% [7/1401]). The medium time from hospital arrival to vessel imaging was 1 day (interquartile range, 0–2.0). The distribution of LVO in ICA, MCA, ACA, PCA, VA, BA were 19.5% (273/1401), 35.7% (500/1401), 34.8% (488/1401), 39.0% (547/1401), 30.3% (425/1401), and 6.4% (90/1401), respectively, and no significant difference was observed among the three groups regarding the distribution of LVO. The medium NIHSS score was 2.0 (interquartile range: 1.0–4.0) for all different LVO locations. The most common reasons for patients without intravenous t‐PA in DAPT and aspirin monotherapy groups were mild symptoms (35.30%), rapid improvement of stroke symptoms (14.61%), and patient or family refusal (17.04%).

**TABLE 1:** Baseline characteristics of patients based on the modalities of medications.

Variables	Total	Intravenous t‐PA	DAPT	Aspirin	*p* value
	(*N* = 1401)	(*N* = 251)	(*N* = 722)	(*N* = 428)	
Age, y	63.0 (56.0–71.0)	62.0 (55.0–68.0)	63.0 (56.0–70.0)	65.0 (55.5–74.0)	0.004
Male	974 (69.5)	178 (70.9)	510 (70.6)	286 (66.8)	0.35
Current smoking	451 (32.2)	89 (35.5)	246 (34.1)	116 (27.1)	0.02
Hypertension	911 (65.0)	166 (66.1)	468 (64.8)	277 (64.7)	0.92
Diabetes mellitus	354 (25.3)	52 (20.7)	211 (29.2)	91 (21.3)	0.002
Dyslipidemia	116 (8.3)	21 (8.4)	61 (8.4)	34 (7.9)	0.95
Prior CHD/MI	144 (10.3)	19 (7.6)	73 (10.1)	52 (12.1)	0.16
Atrial fibrillation	40 (2.9)	14 (5.6)	11 (1.5)	15 (3.5)	0.003
Prior TIA	37 (2.6)	3 (1.2)	25 (3.5)	9 (2.1)	0.11
Prior stroke	307 (21.9)	44 (17.5)	169 (23.4)	94 (22.0)	0.15
Random blood glucose, mmol/L	7.1 (5.9–8.9)	6.0 (5.6–7.6)	7.4 (6.1–12.2)	7.2 (6.0–8.9)	0.15
Baseline SBP, mmHg	149.5 (137.0–163.5)	151.0 (140.0–167.0)	148.3 (136.5–162.0)	150.0 (136.3–165.0)	0.13
Baseline DBP, mmHg	85.0 (79.0–95.0)	88.0 (79.0–96.5)	85.0 (79.0–95.0)	85.0 (78.8–95.0)	0.19
Weight, kg	70.0 (61.0–75.0)	69.0 (60.0–75.0)	70.0 (62.0–76.0)	70.0 (62.3–75.0)	0.53
Pre‐mRS 0–1	1289 (92.0)	237 (94.4)	653 (90.4)	399 (93.2)	0.07
Baseline NIHSS	2.0 (1.0–4.0)	3.0 (2.0–4.0)	2.0 (1.0–4.0)	2.0 (1.0–4.0)	<0.001
Care in stroke unit	317 (22.6)	111 (44.2)	137 (19.0)	69 (16.1)	<0.001
TOAST subtype					0.15
LAA	668 (47.7)	119 (47.4)	357 (49.4)	192 (44.9)	
Cardioembolic	57 (4.1)	17 (6.8)	21 (2.9)	19 (4.4)	
SAO	239 (17.0)	41 (16.3)	120 (16.6)	78 (18.2)	
Other determined cause	32 (2.3)	6 (2.4)	20 (2.8)	6 (1.4)	
Undetermined cause	405 (28.9)	68 (27.1)	204 (28.3)	133 (31.1)	
Site of occlusion*					
ICA	273 (19.5)	44 (17.5)	145 (20.1)	84 (19.6)	0.68
MCA	500 (35.7)	79 (31.5)	261 (36.1)	160 (37.4)	0.28
ACA	488 (34.8)	83 (33.1)	262 (36.3)	143 (33.4)	0.50
PCA	547 (39.0)	91 (36.3)	280 (38.8)	176 (41.1)	0.45
VA	425 (30.3)	79 (31.5)	217 (30.1)	129 (30.1)	0.91
BA	90 (6.4)	14 (5.6)	57 (7.9)	19 (4.4)	0.06
Medication history					
Antiplatelet	220 (15.7)	23 (9.2)	129 (17.9)	68 (15.9)	0.005
Anticoagulant	9 (0.6)	2 (0.8)	4 (0.6)	3 (0.7)	0.90
Lipid lowering agents	146 (10.4)	15 (6.0)	87 (12.0)	44 (10.3)	0.03
Laboratory findings					
Hemoglobin, g/L	142.0 (132.0–152.0)	144.0 (134.0–153.0)	142.0 (132.0–152.0)	140.0 (131.0–152.0)	0.26
Platelet count, ×10^9^/L	209.0 (174.3–249.0)	214.0 (180.0–253.0)	208.0 (174.0–247.0)	204.0 (171.0–248.7)	0.24
Serum creatinine, μmol/L	71.0 (59.5–83.0)	69.0 (59.0–78.0)	71.0 (59.0–84.0)	71.0 (60.0–83.0)	0.28
LDL cholesterol, mmol/L	2.4 (1.9–3.0)	2.4 (1.8–2.9)	2.5 (1.9–3.1)	2.4 (1.9–3.0)	0.44
Time measures, hours					
Onset to door	2.0 (1.0–3.0)	1.8 (1.0–2.4)	2.0 (1.0–3.1)	2.1 (0.8–3.3)	<0.001
Onset to needle	2.9 (2.1–3.7)	2.9 (2.1–3.7)	—	—	—
Door to needle	1.0 (0.5–1.6)	1.0 (0.5–1.6)	—	—	—

*Note*: Values are presented as median (interquartile range) or as No. (%). DAPT indicates dual antiplatelet treatment; t‐PA, tissue‐type plasminogen activator; CHD, coronary heart disease; MI, myocardial infarction; TIA, transient ischemic attack; SBP, systolic blood pressure; DBP, diastolic blood pressure; mRS, modified Rankin Scale; NIHSS, National Institutes of Health Stroke Scale; TOAST, Trial of ORG 10172 in Acute Stroke Treatment; LAA, large artery atherosclerosis; SAO, small artery occlusion; ICA, internal carotid artery; MCA, middle cerebral artery; ACA, anterior cerebral artery; PCA, posterior cerebral artery; VA, vertebral artery; BA, basilar artery; LDL, low density lipoprotein.

* The sum of the LVO distributions for each group did not equal 100% because of the combined phenomena.

### 3.3 Functional outcome

Our analysis showed that the proportion of excellent functional outcome was significantly higher in intravenous t‐PA group (86.80% [217/250]) than in DAPT group (82.94% [593/715]) and aspirin group (77.20% [325/421]) (DAPT versus intravenous t‐PA; adjusted odds ratio [aOR], 0.76; 95% CI, 0.49 to 1.19; *p* = 0.23; aspirin versus intravenous t‐PA; aOR, 0.50; 95% CI, 0.32 to 0.80; *p* = 0.004, respectively) (Table 2). The proportions of favorable functional outcome in intravenous t‐PA, DAPT, and aspirin groups were 94.80% (237/250), 93.15% (666/715), 90.02% (379/421), respectively, and intravenous t‐PA was associated with favorable functional outcome compared with aspirin alone before adjusting potential covariates (OR, 0.49; 95% CI, 0.26 to 0.94; *p* = 0.03). Nevertheless, after adjusting covariates, no treatment effect was demonstrated among three groups (DAPT versus intravenous t‐PA, aOR, 0.85; 95% CI, 0.44 to 1.65; *p* = 0.63; aspirin versus intravenous t‐PA; aOR, 0.59; 95% CI, 0.30 to 1.17; *p* = 0.13, respectively) (Table 2). The results were consistent with those using PSM analysis (Tables S1–S4).

**TABLE 2:** Comparison of main outcomes in patients with three different modalities of medications.

Outcome	Group	Event/N (%)	Unadjusted		Multivariable adjusted*	
			OR (95%CI)	*p* value	OR (95%CI)	*p* value
mRS 0–1 at 90 days	Intravenous t‐PA	217/250 (86.80)	Ref.		Ref.	
	DAPT	593/715 (82.94)	0.74 (0.49, 1.12)	0.15	0.76 (0.49, 1.19)	0.23
	Aspirin	325/421 (77.20)	0.51 (0.33, 0.79)	0.003	0.50 (0.32, 0.80)	0.004
mRS 0–2 at 90 days	Intravenous t‐PA	237/250 (94.80)	Ref.		Ref.	
	DAPT	666/715 (93.15)	0.75 (0.40, 1.40)	0.36	0.85 (0.44, 1.65)	0.63
	Aspirin	379/421 (90.02)	0.49 (0.26, 0.94)	0.03	0.59 (0.30, 1.17)	0.13
Recurrent stroke at 90 days	Intravenous t‐PA	19/251 (7.57)	Ref.		Ref.	
	DAPT	51/722 (7.06)	0.93 (0.54, 1.60)	0.79	0.88 (0.49, 1.57)	0.67
	Aspirin	29/428 (6.78)	0.89 (0.49, 1.62)	0.70	0.88 (0.47, 1.66)	0.70
Recurrent ischemic stroke at 90 days	Intravenous t‐PA	18/251 (7.17)	Ref.		Ref.	
	DAPT	51/722 (7.06)	0.98 (0.56, 1.72)	0.95	0.94 (0.52, 1.70)	0.85
	Aspirin	29/428 (6.78)	0.94 (0.51, 1.73)	0.84	0.94 (0.50, 1.79)	0.85
All‐cause mortality at 90 days	Intravenous t‐PA	0/251 (0)	Ref.		Ref.	
	DAPT	4/722 (0.55)	—	—	—	—
	Aspirin	10/428 (2.34)	—	—	—	—

Abbreviations: DAPT, dual antiplatelet treatment; mRS indicates modified Rankin Scale; NIHSS, National Institutes of Health Stroke Scale; t‐PA, tissue‐type plasminogen activator.

^a^ Adjusted for age, current smoking, diabetes mellitus, atrial fibrillation, pre‐mRS, baseline NIHSS, site of vessel occlusion, onset to door time, care in stroke unit, medication history of antiplatelet and lipid‐lowering agents.

### 3.4 Recurrent stroke

There was no statistical difference in 90‐day recurrent stroke among three groups (7.06% [51/722] DAPT versus 7.57% [19/251] intravenous t‐PA; aOR, 0.88; 95% CI, 0.49 to 1.57; *p* = 0.67; 6.78% [29/428] aspirin versus 7.57% [19/251] intravenous t‐PA; aOR, 0.88; 95% CI, 0.47 to 1.66; *p* = 0.70, respectively) (Table 2). Similar results were detected for 90‐day recurrent ischemic stroke (7.06% [51/722] DAPT versus 7.17% [18/251] intravenous t‐PA; aOR, 0.94; 95% CI, 0.52 to 1.70; *p* = 0.85; 6.78% [29/428] aspirin versus 7.17% [18/251] intravenous t‐PA; aOR, 0.94; 95% CI, 0.50 to 1.79; *p* = 0.85, respectively) (Table 2). The 90‐day Kaplan–Meier survival analyses for recurrent stroke and ischemic stroke are shown in Figures (S1 and S2).

### 3.5 All‐cause mortality

There were 4 patients (0.55%) died at 90 days in the DAPT group, 10 (2.34%) in the aspirin group, and none in the intravenous t‐PA group (Table 2).

### 3.6 Hemorrhagic events

The hemorrhagic safety outcomes are shown in Table S5. No patient developed sICH or severe systemic hemorrhage complications within 36 h of intravenous t‐PA.

### 3.7 Subgroup analysis of disabling versus non‐disabling stroke

**FIGURE 2:**
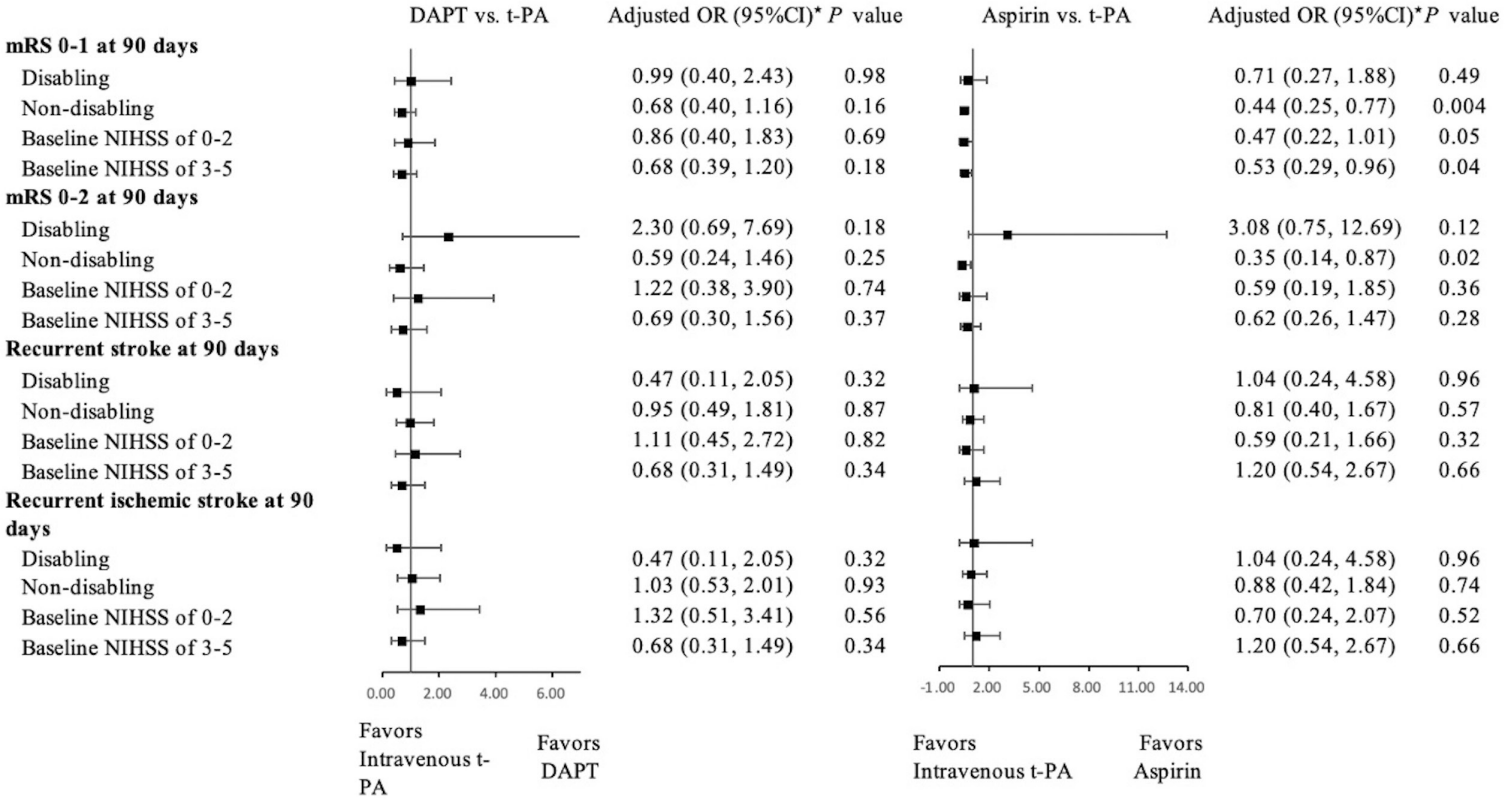
Subgroup analysis. *Adjusted for age, current smoking, diabetes mellitus, atrial fibrillation, pre‐mRS, baseline NIHSS, site of vessel occlusion, onset to door time, care in stroke unit, medication history of antiplatelet and lipid‐lowering agents. mRS indicates modified Rankin Scale; NIHSS, National Institutes of Health Stroke Scale; DAPT, dual antiplatelet treatment; t‐PA, tissue‐type plasminogen activator.

In non‐disabling subgroup, patients treated with intravenous t‐PA were associated with higher odds for excellent and favorable functional outcomes compared with patients with aspirin alone (aspirin versus intravenous t‐PA; aOR, 0.44; 95% CI, 0.25 to 0.77; *p* = 0.004; aOR, 0.35; 95% CI, 0.14 to 0.87; *p* = 0.02, respectively) (Table 3, Figure 2). The recurrent stroke and recurrent ischemic stroke were not significantly different among the three groups (Figure 2). In disabling subgroup, the excellent functional outcome, favorable functional outcome, recurrent stroke, and recurrent ischemic stroke were not significantly different among the three groups (Figure 2).

**TABLE 3:** Proportions of the main outcome events of subgroups.

Group		Event/*N* (%)	
		mRS 0–1 at 90 days	mRS 0–2 at 90 days
Disabling	Intravenous t‐PA	38/51 (74.51)	44/51 (86.27)
	DAPT	67/99 (67.68)	84/99 (84.85)
	Aspirin	36/59 (61.02)	51/59 (86.44)
Non‐disabling	Intravenous t‐PA	179/199 (89.95)	193/199(96.98)
	DAPT	526/616 (85.39)	582/616 (94.48)
	Aspirin	289/362 (79.83)	328/362 (90.61)
Baseline NIHSS of 0–2	Intravenous t‐PA	102/112 (91.07)	108/112 (96.43)
	DAPT	353/396 (89.14)	381/396 (96.21)
	Aspirin	198/241 (82.16)	221/241 (91.70)
Baseline NIHSS of 3–5	Intravenous t‐PA	115/138 (83.33)	129/138 (93.48)
	DAPT	240/319 (75.24)	285/319 (89.34)
	Aspirin	127/180 (70.56)	158/180 (87.78)

Abbreviations: DAPT, dual antiplatelet treatment; mRS, modified Rankin Scale; NIHSS, National Institutes of Health Stroke Scale; t‐PA, tissue‐type plasminogen activator.

### 3.8 Subgroup analysis of baseline NIHSS of 0–2 versus NIHSS of 3–5

In baseline NIHSS score of 3 to 5 subgroups, patients treated with intravenous t‐PA were associated with higher odds for excellent functional outcome compared with patients with aspirin alone (aspirin versus intravenous t‐PA; 70.56% vs. 83.33%; aOR, 0.53; 95% CI, 0.29 to 0.96; *p* = 0.04) (Table 3, Figure 2). The favorable functional outcome, recurrent stroke, and recurrent ischemic stroke were not significantly different among the three groups (Figure 2). In baseline NIHSS score of 0 to 2 subgroups, although both the rates of excellent and favorable functional outcomes were the highest in intravenous t‐PA group (91.07% [102/112] and 96.43% [108/112], respectively), there was no significant difference among the three groups (Table 3, Figure 2). The recurrent stroke and recurrent ischemic stroke were not significantly different among the three groups (Figure 2).

### 3.9 Symptomatic LVO

In the subgroup of symptomatic LVO, the proportions of 90‐day mRS score of 0 to 1 in intravenous t‐PA, DAPT, and aspirin groups were 85.51% (118/138), 80.45% (321/399), and 75.34% (165/219), respectively (DAPT versus intravenous t‐PA; aOR, 0.73; 95% CI, 0.41 to 1.31; *p* = 0.30; aspirin versus intravenous t‐PA; aOR, 0.50; 95% CI, 0.27 to 0.93; *p* = 0.03, respectively) (Tables S6 and S7). Using PSM analysis, the results were similar (Tables S8–S11).

### 3.10 Asymptomatic LVO

In the subgroup of asymptomatic LVO, the rates of 90‐day mRS score of 0 to 1 in intravenous t‐PA, DAPT, and aspirin groups were 88.39% (99/112), 86.08% (272/316), 79.21% (160/202), respectively (DAPT versus intravenous t‐PA; aOR, 0.72; 95% CI, 0.35 to 1.47; *p* = 0.36; aspirin versus intravenous t‐PA; aOR, 0.47; 95% CI, 0.22 to 0.98; *p* = 0.045, respectively) (Tables S12 and S13).

## 4 DISCUSSION

Our study found that intravenous t‐PA with minor stroke and LVO was better than aspirin, but similar to DAPT for achieving 90‐day excellent functional outcome. Sensitivity analysis based on LVO symptomatology showed similar results. In addition, intravenous t‐PA had no sICH and severe systemic bleeding within 36 h.

Prior studies have shown that minor stroke could be associated with high odds of disability and the risk would increase in patients with LVO. Our data showed the overall rate of severe functional impairment (mRS = 3–6) was around 7% in three groups and a 18% rate of unfavorable functional impairment (mRS = 2–6). These are slightly lower than those (10% and 34%) in the TNK‐tPA Evaluation for Minor Ischemic Stroke with Proven Occlusion (TEMPO‐1) trial,^20^ and it might be explained by the less deficit severity of the patients included in our study (median baseline NIHSS of 2.0 in our study versus 2.5 in TEMPO‐1) and different ethnicity.

The efficacy and safety of intravenous thrombolysis in patients with minor stroke harboring LVO were not fully demonstrated in prior studies. The TEMPO‐1 trial^20^ was a phase 2, dose‐escalated trial and demonstrated the feasibility and safety of tenecteplase as another alternative thrombolysis treatment in patients with minor stroke and LVO. And the ongoing TEMPO‐2 trial (NCT02398656) compares tenecteplase to standard antiplatelet therapy in patients with minor stroke and LVO. Our study documented an independent association of intravenous t‐PA with higher odds of 90‐day excellent functional outcome in multivariable models after adjustment for confounders compared with aspirin group. However, when comparing with DAPT group, intravenous t‐PA was not associated with better 90‐day excellent and favorable outcome. Our findings were consistent with a recent analysis of 103,765 United States minor stroke (NIHSS ≤5) inpatients cohort which also found t‐PA was associated with the increased excellent functional outcome.^21^ A single‐center cohort of 185 patients showed that primary intravenous thrombolysis was associated with better 90‐day excellent outcome compared with primary conservative therapy.^10^ In addition, another multicenter, international cohort of 336 minor stroke patients attributed to LVO or distal occlusions reported that t‐PA was associated with a higher likelihood of the 90‐day excellent and favorable outcomes.^22^ Up to now, head‐to‐head comparisons between thrombolytic drugs and antiplatelet agents are also lacking. Some ongoing trials such as Antiplatelet vs R‐tPA for Acute Mild Ischemic Stroke (ARAMIS) (NCT03661411) and TEMPO‐2 (NCT02398656) will provide more evidence.

In the present analysis, there was no 36‐h sICH and severe systemic bleeding in the intravenous t‐PA group. Although the safety outcomes among the three groups were incomparable, the risk of hemorrhagic events in our patients who received intravenous t‐PA was consistent with previous studies (0% to 5%).^12^, ^22^, ^23^, ^24^

In subgroup analysis, the effect of intravenous t‐PA was statistically more pronounced in the non‐disabling than in disabling subgroup. This is in contrast to the PRISMS trial which indicated that alteplase was not superior to the aspirin in improving functional outcomes in non‐disabling minor stroke.^12^ But this trial did not reach prespecified sample size and was underpowered to make a strong conclusion. In our study, our definitions of disabling and non‐disabling minor stroke were based on NIHSS subitems and it was different from the criteria in PRISMS in which clearly disabling deficits were defined as preventing patients return to work or perform basic activities of daily living. Another small cohort of 461 patients reported a 28.7% difference of the rate of 90‐day excellent outcome between intravenous alteplase and no‐treated alteplase groups in patients with minor non‐disabling stroke and severe stenosis/LVO. It showed that patients with such characteristics could benefit from intravenous alteplase. In the study, minor non‐disabling stroke was defined as patients with baseline NIHSS score ≤5 and a score of 0 or 1 on each baseline NIHSS score item (items 1a to 1c being 0).^25^ However, these findings need to be verified in future randomized trials. We did not find a significant association between intravenous t‐PA and excellent outcome in disabling subgroup. One possible explanation for this result is the small sample size in disabling subgroup. According to the current rates of excellent outcome in disabling subgroup (74.51 [38/51] in intravenous t‐PA, 67.68 [67/99] in DAPT, and 61.02 [36/59] in aspirin), for yielding 80% power, an estimated total sample of 695 patients will be needed at two‐sided alpha of 0.05.

A similar effect happened between baseline NIHSS of 3 to 5 and 0 to 2 subgroup. The effect of intravenous t‐PA was statistically more pronounced in baseline NIHSS of the 3 to 5 than in the 0 to 2 subgroup. It was in line with the Mild and Rapidly Improving Stroke Study (MaRISS).^26^, ^27^ MaRISS study included patients with minor stroke (NIHSS≤5) and TIA from the American Get With The Guidelines‐Stroke registry and identified a better 90‐day Stroke Impact Scale‐16 (SIS‐16) in the baseline NIHSS score 3 to 5 subgroup, but the study did not incorporate imaging markers. Regarding baseline NIHSS score 0 to 2 subgroup, although the rate of excellent and favorable functional outcomes in intravenous t‐PA was higher compared to those of DAPT and aspirin groups, there were no significant differences detected. It might be that effect size was underpowered on the mRS outcome in this subgroup.

Our study has several limitations. First, it is a cohort study but not a randomized controlled trial. However, the CNSR‐III was a multicenter prospective registry with broad representative of clinical practice for patients with baseline low NIHSS with LVO. Further randomized controlled trials are warranted. Second, the small number of cases in the intravenous t‐PA group limited power to estimate the effect size among subgroups, and the excellent functional outcome only showed a better trend in patients treated with intravenous t‐PA in disabling subgroup. Third, data on 36‐h sICH and severe systemic bleeding in the DAPT and aspirin group were missing in our registry, making it difficult to compare the key safety outcome of these strategies. Fourth, imaging evaluation was not standardized in our study, LVO was detected by either MRA, CTA, or DSA, and the imaging to onset time was about 1 day, which means some patients with t‐PA may have LVO imaging screening after t‐PA usage, so we may miss those recanalized patients after t‐PA. Therefore, our study only included patients with t‐PA and LVO may underestimate the effectiveness of t‐PA. However, it will not change our findings that t‐PA is better than aspirin. Whether t‐PA is superior to DAPT is still waiting for further investigations. In addition, due to inadequate drug compliance, there was a certain rate in patients with discontinuation of antiplatelet therapy during follow‐up. Our study is only exploratory analysis based on the registry cohort, and the results need to be verified in other multicenter prospective cohorts, preferably randomized controlled trials.

## 5 CONCLUSION

Minor stroke harboring LVO receiving intravenous t‐PA, as compared with aspirin monotherapy, is associated with higher odds for 90‐day excellent functional outcome. Further randomized controlled trials are needed.

## AUTHOR CONTRIBUTIONS

Chunmiao Duan and Yunyun Xiong contributed equally. Manuscript draft and editorial design: Chunmiao Duan and Yunyun Xiong. Statistical analysis: Hongqiu Gu and Kai‐Xuan Yang. Critical revision of the manuscript: Shang Wang, Manjun Hao and Xueyan Feng. Project supervision: Xingquan Zhao and Mia Meng. Editorial design and funding acquisition: Yongjun Wang.

## FUNDING INFORMATION

This work was supported by grants from the Capital's Funds for Health Improvement and Research (2020–1‐2041), Chinese Academy of Medical Sciences Innovation Fund for Medical Sciences (2019‐I2M‐5‐029), National Natural Science Foundation of China (81,870,905, U20A20358, 82,171,272), Beijing Municipal Science & Technology Commission (Z211100003521019), Beijing Hospitals Authority (PX2022019).

## CONFLICT OF INTEREST STATEMENT

The authors declare no conflict of interest.

## Supporting information


**Data S1:** Supporting Information

## Data Availability

The data that support the findings of this study are available from the corresponding authors upon reasonable request and with approval from the CNSR‐III investigators.
