# Supplementary material for: Intravenous thrombolysis versus antiplatelet therapy in minor stroke patients with large vessel occlusion
Source: CNS Neurosci Ther. 2023 Mar 7;29(6):1615–23. doi: 10.1111/cns.14124 (PMC10173711; doi:10.1111/cns.14124)
Supplement: Supplementary file 1 — Data S1: Supporting Information [file CNS-29-1615-s001.docx]

**Supplementary Materials**

Supplementary Table 1. Baseline Characteristics of Patients with Aspirin and Intravenous t-PA in Whole Cohort Using Propensity Score Matching

Supplementary Table 2. Comparison of 90-Day Outcomes in Patients with Aspirin and Intravenous t-PA in Whole Cohort Using Propensity Score Matching

Supplementary Table 3. Baseline Characteristics of Patients with DAPT and Intravenous t-PA in Whole Cohort Using Propensity Score Matching

Supplementary Table 4. Comparison of 90-Day Outcomes in Patients with DAPT and Intravenous t-PA in Whole Cohort Using Propensity Score Matching

Supplementary Table 5. Hemorrhagic Events of Patients with Three Different Modalities of Medications

Supplementary Table 6. Baseline Characteristics of Patients in Symptomatic Large Vessel Occlusion

Supplementary Table 7. Main Outcomes in Symptomatic Large Vessel Occlusion

Supplementary Table 8. Baseline Characteristics of Patients with Aspirin and Intravenous t-PA in Symptomatic Large Vessel Occlusion Using Propensity Score Matching

Supplementary Table 9. Comparison of 90-Day Outcomes in Patients with Aspirin and Intravenous t-PA in Symptomatic Large Vessel Occlusion Using Propensity Score Matching

Supplementary Table 10. Baseline Characteristics of Patients with DAPT and Intravenous t-PA in Symptomatic Large Vessel Occlusion Using Propensity Score Matching

Supplementary Table 11. Comparison of 90-Day Outcomes in Patients with DAPT and Intravenous t-PA in Symptomatic Large Vessel Occlusion Using Propensity Score Matching

Supplementary Table 12. Baseline Characteristics of Patients in Asymptomatic Large Vessel Occlusion

Supplementary Table 13. Main Outcomes in Asymptomatic Large Vessel Occlusion

Supplementary Figure 1. Kaplan-Meier Curve for the Recurrent Stroke at 90 Days

Supplementary Figure 2. Kaplan-Meier Curve for the Recurrent Ischemic Stroke at 90 Days

**Supplementary Table 1. Baseline Characteristics of Patients with Aspirin and Intravenous t-PA in Whole Cohort Using Propensity Score Matching**

| Variables | Total | Intravenous  t-PA | Aspirin | | *P* value | |
| --- | --- | --- | --- | --- | --- | --- |
|  | (N=418) | (N=209) | (N=209) | |  |  |
| Age, y | 62.0 (54.0-71.0) | 63.0 (55.0-69.0) | 62.0 (53.0-73.0) | | 0.78 | |
| Male | 290 (69.4) | 145 (69.4) | 145 (69.4) | | 1.00 | |
| Current smoking | 132 (31.6) | 69 (33.0) | 63 (30.1) | | 0.53 | |
| Hypertension | 261 (62.4) | 136 (65.1) | 125 (59.8) | | 0.27 | |
| Diabetes mellitus | 89 (21.3) | 41 (19.6) | 48 (23.0) | | 0.40 | |
| Dyslipidemia | 35 (8.4) | 19 (9.1) | 16 (7.7) | | 0.60 | |
| Prior CHD/MI | 43 (10.3) | 17 (8.1) | 26 (12.4) | | 0.15 | |
| Atrial fibrillation | 21 (5.0) | 11 (5.3) | 10 (4.8) | | 0.82 | |
| Prior TIA | 7 (1.7) | 3 (1.4) | 4 (1.9) | | 0.70 | |
| Prior Stroke | 79 (18.9) | 36 (17.2) | 43 (20.6) | | 0.38 | |
| Random blood glucose, mmol/L | 7.0 (5.7-9.1) | 6.0 (5.6-7.6) | 7.4 (6.3-9.1) | | 0.24 | |
| Baseline SBP, mmHg | 150.0 (136.5-165.0) | 151.0 (139.0-165.0) | 147.5 (132.0-163.0) | | 0.17 | |
| Baseline DBP, mmHg | 85.0 (78.0-93.5) | 87.0 (78.0-95.5) | 84.0 (78.0-92.0) | | 0.12 | |
| Weight, kg | 70.0 (61.0-75.0) | 69.0 (60.0-75.0) | 70.0 (63.0-76.0) | | 0.18 | |
| Pre-mRS 0-1 | 391 (93.5) | 197 (94.3) | 194 (92.8) | | 0.55 | |
| Baseline NIHSS | 2.0 (1.0-4.0) | 3.0 (1.0-4.0) | 2.0 (1.0-4.0) | | 0.12 | |
| Care in stroke unit | 128 (30.6) | 69 (33.0) | 59 (28.2) | | 0.29 | |
| TOAST subtype |  |  |  | | 0.83 | |
| LAA | 202 (48.3) | 103 (49.3) | 99 (47.4) | |  | |
| Cardioembolic | 23 (5.5) | 13 (6.2) | 10 (4.8) | |  | |
| SAO | 62 (14.8) | 32 (15.3) | 30 (14.4) | |  | |
| Other determined cause | 9 (2.2) | 5 (2.4) | 4 (1.9) | |  | |
| Undetermined cause | 122 (29.2) | 56 (26.8) | 66 (31.6) | |  | |
| Site of occlusion* |  |  |  |  | |  |
| ICA | 80 (19.1) | 36 (17.2) | 44 (21.1) | 0.32 | |  |
| MCA | 159 (38.0) | 71 (34.0) | 88 (42.1) | 0.09 | |  |
| ACA | 129 (30.9) | 67 (32.1) | 62 (29.7) | 0.60 | |  |
| PCA | 164 (39.2) | 75 (35.9) | 89 (42.6) | 0.16 | |  |
| VA | 126 (30.1) | 66 (31.6) | 60 (28.7) | 0.52 | |  |
| BA | 24 (5.7) | 11 (5.3) | 13 (6.2) | 0.67 | |  |
| Medication history |  |  |  | |  | |
| Antiplatelet | 51 (12.2) | 21 (10.0) | 30 (14.4) | | 0.18 | |
| Anticoagulant | 4 (1.0) | 2 (1.0) | 2 (1.0) | | 1.00 | |
| Lipid lowering agents | 35 (8.4) | 15 (7.2) | 20 (9.6) | | 0.38 | |
| Laboratory findings |  |  |  | |  | |
| Hemoglobin, g/L | 142.0 (133.0-154.0) | 144.0 (134.0-153.0) | 141.0 (131.5-154.0) | | 0.55 | |
| Platelet count, ×10^9^/L | 210.0 (174.0-248.0) | 211.0 (177.0-244.0) | 207.5 (172.0-252.0) | | 0.96 | |
| Serum creatinine, μmol/L | 69.0 (59.0-81.0) | 69.0 (59.0-79.0) | 68.1 (59.7-85.5) | | 0.57 | |
| LDL cholesterol, mmol/L | 2.4 (1.8-3.0) | 2.4 (1.8-3.0) | 2.4 (1.9-3.0) | | 0.86 | |

Values are presented as median (interquartile range) or as No. (%). DAPT indicates dual antiplatelet treatment; t-PA, tissue-type plasminogen activator; CHD, coronary heart disease; MI, myocardial infarction; TIA, transient ischemic attack; SBP, systolic blood pressure; DBP, diastolic blood pressure; mRS, modified Rankin Scale; NIHSS, National Institutes of Health Stroke Scale; TOAST, Trial of ORG 10172 in Acute Stroke Treatment; LAA, large artery atherosclerosis; SAO, small artery occlusion; ICA, internal carotid artery; MCA, middle cerebral artery; ACA, anterior cerebral artery; PCA, posterior cerebral artery; VA, vertebral artery; BA, basilar artery; LDL, low density lipoprotein.

*The sum of the LVO distributions for each group did not equal 100% because of the combined phenomena.

**Supplementary Table 2. Comparison of 90-Day Outcomes in Patients with Aspirin and Intravenous t-PA in Whole Cohort Using Propensity Score Matching**

| Group | Outcome | Aspirin/t-PA  (%) | OR (95%CI) | *P* value |
| --- | --- | --- | --- | --- |
|  |  |  |  |  |
| All | mRS 0-1 | 77.94/86.54 | 0.55 (0.33, 0.92) | 0.02 |
|  | mRS 0-2 | 90.20/95.19 | 0.46 (0.21, 1.02) | 0.06 |
|  | Recurrent stroke | 9.09/7.66 | 1.21 (0.60, 2.42) | 0.60 |
|  | Recurrent ischemic stroke | 9.09/7.18 | 1.29 (0.64, 2.62) | 0.48 |
|  | All-cause mortality | 1.91/0.00 | - | - |
| Disabling | mRS 0-1 | 64.29/74.36 | 0.62 (0.22, 1.78) | 0.38 |
|  | mRS 0-2 | 89.29/87.18 | 1.23 (0.27, 5.61) | 0.79 |
|  | Recurrent stroke | 14.29/7.69 | 2.00 (0.41, 9.74) | 0.39 |
|  | Recurrent ischemic stroke | 14.29/7.69 | 2.00 (0.41, 9.74) | 0.39 |
|  | All-cause mortality | 0.00/0.00 | - | - |
| Non-disabling | mRS 0-1 | 80.11/89.35 | 0.48 (0.26, 0.89) | 0.02 |
|  | mRS 0-2 | 90.34/97.04 | 0.29 (0.10, 0.79) | 0.02 |
|  | Recurrent stroke | 8.29/7.65 | 1.09 (0.50, 2.37) | 0.82 |
|  | Recurrent ischemic stroke | 8.29/7.06 | 1.19 (0.54, 2.62) | 0.67 |
|  | All-cause mortality | 2.21/0.00 | - | - |
| Baseline NIHSS of 0-2 | mRS 0-1 | 83.04/91.00 | 0.48 (0.21, 1.13) | 0.09 |
|  | mRS 0-2 | 91.07/96.00 | 0.43 (0.13, 1.40) | 0.16 |
|  | Recurrent stroke | 6.14/7.00 | 0.87 (0.29, 2.57) | 0.80 |
|  | Recurrent ischemic stroke | 6.14/6.00 | 1.02 (0.33, 3.16) | 0.97 |
|  | All-cause mortality | 0.88/0.00 | - | - |
| Baseline NIHSS of 3-5 | mRS 0-1 | 71.74/82.41 | 0.54 (0.28, 1.06) | 0.07 |
|  | mRS 0-2 | 89.13/94.44 | 0.48 (0.17, 1.38) | 0.17 |
|  | Recurrent stroke | 12.63/8.26 | 1.61 (0.65, 4.00) | 0.31 |
|  | Recurrent ischemic stroke | 12.63/8.26 | 1.61 (0.65, 4.00) | 0.31 |
|  | All-cause mortality | 3.16/0.00 | - | - |

NIHSS indicates National Institutes of Health Stroke Scale; mRS, modified Rankin Scale; t-PA, tissue-type plasminogen activator.

**Supplementary Table 3. Baseline Characteristics of Patients with DAPT and Intravenous t-PA in Whole Cohort Using Propensity Score Matching**

| Variables | Total | Intravenous  t-PA | DAPT | | *P* value | |
| --- | --- | --- | --- | --- | --- | --- |
|  | (N=472) | (N=236) | (N=236) | |  |  |
| Age, y | 62.0 (55.0-68.0) | 62.0 (55.0-69.0) | 62.0 (55.0-68.0) | | 0.93 | |
| Male | 340 (72.0) | 165 (69.9) | 175 (74.2) | | 0.31 | |
| Current smoking | 167 (35.4) | 84 (35.6) | 83 (35.2) | | 0.92 | |
| Hypertension | 299 (63.3) | 158 (66.9) | 141 (59.7) | | 0.10 | |
| Diabetes mellitus | 130 (27.5) | 52 (22.0) | 78 (33.1) | | 0.01 | |
| Dyslipidemia | 40 (8.5) | 20 (8.5) | 20 (8.5) | | 1.00 | |
| Prior CHD/MI | 37 (7.8) | 19 (8.1) | 18 (7.6) | | 0.86 | |
| Atrial fibrillation | 20 (4.2) | 12 (5.1) | 8 (3.4) | | 0.36 | |
| Prior TIA | 10 (2.1) | 3 (1.3) | 7 (3.0) | | 0.20 | |
| Prior Stroke | 95 (20.1) | 44 (18.6) | 51 (21.6) | | 0.42 | |
| Random blood glucose, mmol/L | 6.8 (5.7-8.9) | 6.0 (5.6-7.6) | 7.7 (5.9-10.6) | | 0.22 | |
| Baseline SBP, mmHg | 150.0 (137.5-165.0) | 151.0 (140.5-167.0) | 149.0 (135.0-161.0) | | 0.05 | |
| Baseline DBP, mmHg | 86.0 (79.0-95.8) | 88.0 (78.8-96.5) | 85.0 (79.0-95.0) | | 0.21 | |
| Weight, kg | 70.0 (60.0-75.0) | 69.0 (60.0-75.0) | 70.0 (62.0-75.0) | | 0.65 | |
| Pre-mRS 0-1 | 440 (93.2) | 222 (94.1) | 218 (92.4) | | 0.46 | |
| Baseline NIHSS | 3.0 (1.0-4.0) | 3.0 (1.0-4.0) | 2.0 (1.0-4.0) | | 0.24 | |
| Care in stroke unit | 186 (39.4) | 97 (41.1) | 89 (37.7) | | 0.45 | |
| TOAST subtype |  |  |  | | 0.58 | |
| LAA | 224 (47.5) | 111 (47.0) | 113 (47.9) | |  | |
| Cardioembolic | 26 (5.5) | 17 (7.2) | 9 (3.8) | |  | |
| SAO | 73 (15.5) | 37 (15.7) | 36 (15.3) | |  | |
| Other determined cause | 12 (2.5) | 6 (2.5) | 6 (2.5) | |  | |
| Undetermined cause | 137 (29.0) | 65 (27.5) | 72 (30.5) | |  | |
| Site of occlusion* |  |  |  |  | |  |
| ICA | 89 (18.9) | 42 (17.8) | 47 (19.9) | 0.56 | |  |
| MCA | 156 (33.1) | 72 (30.5) | 84 (35.6) | 0.24 | |  |
| ACA | 170 (36.0) | 76 (32.2) | 94 (39.8) | 0.08 | |  |
| PCA | 182 (38.6) | 88 (37.3) | 94 (39.8) | 0.57 | |  |
| VA | 141 (29.9) | 76 (32.2) | 65 (27.5) | 0.27 | |  |
| BA | 30 (6.4) | 14 (5.9) | 16 (6.8) | 0.71 | |  |
| Medication history |  |  |  | |  | |
| Antiplatelet | 61 (12.9) | 23 (9.7) | 38 (16.1) | | 0.04 | |
| Anticoagulant | 3 (0.6) | 2 (0.8) | 1 (0.4) | | 0.56 | |
| Lipid lowering agents | 39 (8.3) | 14 (5.9) | 25 (10.6) | | 0.07 | |
| Laboratory findings |  |  |  | |  | |
| Hemoglobin, g/L | 144.0 (133.0-153.0) | 144.0 (134.0-153.0) | 145.0 (133.0-154.0) | | 0.62 | |
| Platelet count, ×10^9^/L | 212.5 (180.0-254.0) | 214.0 (180.0-254.0) | 208.0 (180.0-255.0) | | 0.87 | |
| Serum creatinine, μmol/L | 71.0 (59.3-81.0) | 69.0 (59.6-78.0) | 72.0 (59.0-83.0) | | 0.13 | |
| LDL cholesterol, mmol/L | 2.4 (1.9-3.0) | 2.4 (1.8-2.9) | 2.5 (1.9-3.2) | | 0.08 | |

Values are presented as median (interquartile range) or as No. (%). DAPT indicates dual antiplatelet treatment; t-PA, tissue-type plasminogen activator; CHD, coronary heart disease; MI, myocardial infarction; TIA, transient ischemic attack; SBP, systolic blood pressure; DBP, diastolic blood pressure; mRS, modified Rankin Scale; NIHSS, National Institutes of Health Stroke Scale; TOAST, Trial of ORG 10172 in Acute Stroke Treatment; LAA, large artery atherosclerosis; SAO, small artery occlusion; ICA, internal carotid artery; MCA, middle cerebral artery; ACA, anterior cerebral artery; PCA, posterior cerebral artery; VA, vertebral artery; BA, basilar artery; LDL, low density lipoprotein.

*The sum of the LVO distributions for each group did not equal 100% because of the combined phenomena.

**Supplementary Table 4. Comparison of 90-Day Outcomes in Patients with DAPT and Intravenous t-PA in Whole Cohort Using Propensity Score Matching**

| Group | Outcome | DAPT/t-PA  (%) | OR (95%CI) | *P* value |
| --- | --- | --- | --- | --- |
|  |  |  |  |  |
| All | mRS 0-1 | 81.62/88.09 | 0.60 (0.36, 1.01) | 0.05 |
|  | mRS 0-2 | 91.45/94.89 | 0.58 (0.27, 1.21) | 0.14 |
|  | Recurrent stroke | 4.66/7.63 | 0.59 (0.27, 1.28) | 0.18 |
|  | Recurrent ischemic stroke | 4.66/7.20 | 0.63 (0.29, 1.38) | 0.25 |
|  | All-cause mortality | 1.27/0.00 | - | - |
| Disabling | mRS 0-1 | 64.86/75.00 | 0.62 (0.24, 1.61) | 0.32 |
|  | mRS 0-2 | 83.78/84.09 | 0.98 (0.30, 3.21) | 0.97 |
|  | Recurrent stroke | 2.70/11.36 | 0.22 (0.02, 1.94) | 0.17 |
|  | Recurrent ischemic stroke | 2.70/11.36 | 0.22 (0.02, 1.94) | 0.17 |
|  | All-cause mortality | 0.00/0.00 | - | - |
| Non-disabling | mRS 0-1 | 84.77/91.10 | 0.54 (0.29, 1.02) | 0.06 |
|  | mRS 0-2 | 92.89/97.38 | 0.35 (0.12, 1.00) | 0.049 |
|  | Recurrent stroke | 5.03/6.77 | 0.73 (0.31, 1.70) | 0.46 |
|  | Recurrent ischemic stroke | 5.03/6.25 | 0.79 (0.33, 1.88) | 0.60 |
|  | All-cause mortality | 1.51/0.00 | - | - |
| Baseline NIHSS of 0-2 | mRS 0-1 | 87.39/91.07 | 0.68 (0.29, 1.58) | 0.37 |
|  | mRS 0-2 | 94.96/96.43 | 0.70 (0.19, 2.54) | 0.58 |
|  | Recurrent stroke | 5.00/6.25 | 0.79 (0.26, 2.42) | 0.68 |
|  | Recurrent ischemic stroke | 5.00/5.36 | 0.93 (0.29, 2.97) | 0.90 |
|  | All-cause mortality | 1.67/0.00 | - | - |
| Baseline NIHSS of 3-5 | mRS 0-1 | 75.65/85.37 | 0.53 (0.28, 1.03) | 0.06 |
|  | mRS 0-2 | 87.83/93.50 | 0.50 (0.20, 1.25) | 0.14 |
|  | Recurrent stroke | 4.31/8.87 | 0.46 (0.16, 1.38) | 0.17 |
|  | Recurrent ischemic stroke | 4.31/8.87 | 0.46 (0.16, 1.38) | 0.17 |
|  | All-cause mortality | 0.86/0.00 | - | - |

NIHSS indicates National Institutes of Health Stroke Scale; mRS, modified Rankin Scale; DAPT, dual antiplatelet treatment; t-PA, tissue-type plasminogen activator.

**Supplementary Table 5. Hemorrhagic Events of Patients with Three Different Modalities of Medications**

| Outcome | Intravenous t-PA  n (%) | DAPT  n (%) | Aspirin  n (%) |
| --- | --- | --- | --- |
| sICH within 36 hours | 0 (0) | - | - |
| Severe systemic bleeding within 36 hours | 0 (0) | - | - |

sICH indicates symptomatic intracerebral hemorrhage; DAPT, dual antiplatelet treatment; t-PA, tissue-type plasminogen activator.

**Supplementary Table 6. Baseline Characteristics of Patients in Symptomatic Large Vessel Occlusion**

| Variables | Total | Intravenous  t-PA | DAPT | Aspirin | *P* value |
| --- | --- | --- | --- | --- | --- |
|  | (N=762) | (N=139) | (N=402) | (N=221) |  |
| Age, y | 63.0 (56.0-71.0) | 62.0 (55.0-67.0) | 63.5 (57.0-70.0) | 65.0 (55.0-75.0) | 0.02 |
| Male | 523 (68.6) | 101 (72.7) | 283 (70.4) | 139 (62.9) | 0.08 |
| Current smoking | 230 (30.2) | 51 (36.7) | 127 (31.6) | 52 (23.5) | 0.02 |
| Hypertension | 508 (66.7) | 95 (68.3) | 263 (65.4) | 150 (67.9) | 0.74 |
| Diabetes mellitus | 187 (24.5) | 28 (20.1) | 115 (28.6) | 44 (19.9) | 0.02 |
| Dyslipidemia | 68 (8.9) | 13 (9.4) | 36 (9.0) | 19 (8.6) | 0.97 |
| Prior CHD/MI | 83 (10.9) | 13 (9.4) | 41 (10.2) | 29 (13.1) | 0.43 |
| Atrial fibrillation | 19 (2.5) | 6 (4.3) | 6 (1.5) | 7 (3.2) | 0.14 |
| Prior TIA | 26 (3.4) | 3 (2.2) | 21 (5.2) | 2 (0.9) | 0.01 |
| Prior Stroke | 178 (23.4) | 28 (20.1) | 100 (24.9) | 50 (22.6) | 0.50 |
| Random blood glucose, mmol/L | 7.0 (5.8-8.9) | 6.8 (5.6-15.6) | 7.6 (6.0-11.7) | 6.4 (5.8-8.1) | 0.24 |
| Baseline SBP, mmHg | 148.0 (135.5-162.0) | 148.0 (137.5-165.0) | 147.8 (135.0-160.0) | 148.0 (135.0-165.5) | 0.77 |
| Baseline DBP, mmHg | 85.0 (78.5-95.0) | 87.0 (78.5-95.5) | 85.0 (78.5-94.5) | 85.0 (78.5-95.5) | 0.62 |
| Weight, kg | 70.0 (62.0-75.0) | 69.0 (61.0-77.0) | 70.0 (62.0-76.0) | 70.0 (62.3-75.0) | 0.90 |
| Pre-mRS 0-1 | 697 (91.5) | 132 (95.0) | 359 (89.3) | 206 (93.2) | 0.07 |
| Baseline NIHSS | 2.0 (1.0-4.0) | 3.0 (2.0-4.0) | 2.0 (1.0-4.0) | 2.0 (1.0-4.0) | 0.01 |
| Care in stroke unit | 169 (22.2) | 60 (43.2) | 75 (18.7) | 34 (15.4) | <0.001 |
| TOAST subtype |  |  |  |  | 0.84 |
| LAA | 668 (87.7) | 119 (85.6) | 357 (88.8) | 192 (86.9) |  |
| Other determined cause | 19 (2.5) | 4 (2.9) | 10 (2.5) | 5 (2.3) |  |
| Undetermined cause | 75 (9.8) | 16 (11.5) | 35 (8.7) | 24 (10.9) |  |
| Medication history |  |  |  |  |  |
| Antiplatelet | 119 (15.6) | 11 (7.9) | 79 (19.7) | 29 (13.1) | 0.002 |
| Anticoagulant | 3 (0.4) | 0 (0) | 3 (0.7) | 0 (0) | 0.26 |
| Lipid lowering agents | 82 (10.8) | 8 (5.8) | 57 (14.2) | 17 (7.7) | 0.005 |
| Laboratory findings |  |  |  |  |  |
| Hemoglobin, g/L | 142.0 (132.0-153.0) | 144.0 (133.0-155.0) | 142.0 (131.7-153.0) | 140.0 (130.5-152.7) | 0.23 |
| Platelet count, ×10^9^/L | 210.0 (174.0-254.0) | 215.0 (180.0-258.0) | 205.0 (174.0-251.0) | 210.5 (173.5-258.5) | 0.41 |
| Serum creatinine, μmol/L | 71.0 (59.5-84.0) | 69.0 (56.5-77.5) | 72.0 (59.0-85.0) | 69.0 (58.0-85.0) | 0.06 |
| LDL cholesterol, mmol/L | 2.4 (1.9-3.0) | 2.4 (1.8-3.0) | 2.5 (1.9-3.0) | 2.5 (2.0-3.2) | 0.54 |

Values are presented as median (interquartile range) or as No. (%). DAPT indicates dual antiplatelet treatment; t-PA, tissue-type plasminogen activator; CHD, coronary heart disease; MI, myocardial infarction; TIA, transient ischemic attack; SBP, systolic blood pressure; DBP, diastolic blood pressure; mRS, modified Rankin Scale; NIHSS, National Institutes of Health Stroke Scale; TOAST, Trial of ORG 10172 in Acute Stroke Treatment; LAA, large artery atherosclerosis; LDL, low density lipoprotein.

**Supplementary Table 7. Main Outcomes in Symptomatic Large Vessel Occlusion**

| Outcome | Group | Event/N (%) | Unadjusted | |  | Multivariable adjusted* | |
| --- | --- | --- | --- | --- | --- | --- | --- |
|  |  |  | OR (95%CI) | *P* value |  | OR (95%CI) | *P* value |
| mRS 0-1 at 90 days | Intravenous t-PA | 118/138 (85.51) | Ref. |  |  | Ref. |  |
|  | DAPT | 321/399 (80.45) | 0.70 (0.41, 1.19) | 0.19 |  | 0.73 (0.41, 1.31) | 0.30 |
|  | Aspirin | 165/219 (75.34) | 0.52 (0.29, 0.91) | 0.02 |  | 0.50 (0.27, 0.93) | 0.03 |
| mRS 0-2 at 90 days | Intravenous t-PA | 127/138 (92.03) | Ref. |  |  | Ref. |  |
|  | DAPT | 363/399 (90.98) | 0.87 (0.43, 1.77) | 0.71 |  | 0.98 (0.46, 2.08) | 0.95 |
|  | Aspirin | 191/219 (87.21) | 0.59 (0.28, 1.23) | 0.16 |  | 0.70 (0.32, 1.55) | 0.39 |
| Recurrent stroke at 90 days | Intravenous t-PA | 13/139 (9.35) | Ref. |  |  | Ref. |  |
|  | DAPT | 42/402 (10.45) | 1.13 (0.59, 2.18) | 0.71 |  | 1.13 (0.57, 2.27) | 0.73 |
|  | Aspirin | 19/221 (8.6) | 0.91 (0.44, 1.91) | 0.81 |  | 0.76 (0.34, 1.68) | 0.50 |
| Recurrent ischemic stroke at 90 days | Intravenous t-PA | 12/139 (8.63) | Ref. |  |  | Ref. |  |
|  | DAPT | 42/402 (10.45) | 1.23 (0.63, 2.42) | 0.54 |  | 1.25 (0.61, 2.55) | 0.54 |
|  | Aspirin | 19/221 (8.6) | 1.00 (0.47, 2.12) | 0.99 |  | 0.84 (0.37, 1.88) | 0.67 |
| All-cause mortality at 90 days | Intravenous t-PA | 0/139 (0) | Ref. |  |  | Ref. |  |
|  | DAPT | 3/402 (0.75) | - | - |  | - | - |
|  | Aspirin | 8/221 (3.62) | - | - |  | - | - |

*Adjusted for age, sex, current smoking, diabetes mellitus, atrial fibrillation, prior TIA, pre-mRS, baseline NIHSS, care in stroke unit, medication history of antiplatelet and lipid-lowering agents, and serum creatinine.

TIA indicates transient ischemic attack; mRS, modified Rankin Scale; NIHSS, National Institutes of Health Stroke Scale; DAPT, dual antiplatelet treatment; t-PA, tissue-type plasminogen activator.

**Supplementary Table 8. Baseline Characteristics of Patients with Aspirin and Intravenous t-PA in Symptomatic Large Vessel Occlusion Using Propensity Score Matching**

| Variables | Total | Intravenous  t-PA | Aspirin | *P* value |
| --- | --- | --- | --- | --- |
|  | (N=220) | (N=110) | (N=110) |  |
| Age, y | 62.0 (54.0-70.0) | 62.0 (55.0-68.0) | 62.0 (53.0-73.0) | 0.34 |
| Male | 151 (68.6) | 77 (70.0) | 74 (67.3) | 0.66 |
| Current smoking | 67 (30.5) | 39 (35.5) | 28 (25.5) | 0.11 |
| Hypertension | 145 (65.9) | 75 (68.2) | 70 (63.6) | 0.48 |
| Diabetes mellitus | 45 (20.5) | 23 (20.9) | 22 (20.0) | 0.87 |
| Dyslipidemia | 18 (8.2) | 11 (10.0) | 7 (6.4) | 0.33 |
| Prior CHD/MI | 27 (12.3) | 11 (10.0) | 16 (14.5) | 0.30 |
| Atrial fibrillation | 10 (4.5) | 5 (4.5) | 5 (4.5) | 1.00 |
| Prior TIA | 2 (0.9) | 1 (0.9) | 1 (0.9) | 1.00 |
| Prior Stroke | 46 (20.9) | 22 (20.0) | 24 (21.8) | 0.74 |
| Random blood glucose, mmol/L | 6.9 (5.6-8.2) | 6.8 (5.6-15.6) | 7.2 (5.8-8.1) | 0.95 |
| Baseline SBP, mmHg | 149.0 (136.3-165.0) | 150.0 (140.0-165.0) | 148.3 (131.0-166.5) | 0.56 |
| Baseline DBP, mmHg | 86.8 (78.5-96.5) | 86.8 (78.0-96.5) | 86.8 (79.0-96.0) | 0.69 |
| Weight, kg | 70.0 (61.5-76.0) | 69.5 (61.0-77.0) | 70.0 (64.0-76.0) | 0.89 |
| Pre-mRS 0-1 | 207 (94.1) | 103 (93.6) | 104 (94.5) | 0.77 |
| Baseline NIHSS | 3.0 (1.0-4.0) | 3.0 (1.0-4.0) | 3.0 (1.0-4.0) | 0.44 |
| Care in stroke unit | 59 (26.8) | 31 (28.2) | 28 (25.5) | 0.65 |
| TOAST subtype |  |  |  | 0.98 |
| LAA | 191 (86.8) | 95 (86.4) | 96 (87.3) |  |
| Other determined cause | 6 (2.7) | 3 (2.7) | 3 (2.7) |  |
| Undetermined cause | 23 (10.5) | 12 (10.9) | 11 (10.0) |  |
| Medication history |  |  |  |  |
| Antiplatelet | 25 (11.4) | 11 (10.0) | 14 (12.7) | 0.52 |
| Lipid lowering agents | 16 (7.3) | 6 (5.5) | 10 (9.1) | 0.30 |
| Laboratory findings |  |  |  |  |
| Hemoglobin, g/L | 142.5 (132.0-155.0) | 145.0 (133.0-155.0) | 140.0 (130.5-153.5) | 0.15 |
| Platelet count, ×10^9^/L | 215.5 (185.0-259.0) | 215.0 (187.0-258.0) | 217.5 (182.0-261.0) | 0.76 |
| Serum creatinine, μmol/L | 68.1 (56.0-79.0) | 69.0 (56.0-79.0) | 66.7 (55.0-79.0) | 0.67 |
| LDL cholesterol, mmol/L | 2.4 (1.9-3.1) | 2.4 (1.9-3.0) | 2.4 (2.0-3.1) | 0.73 |

Values are presented as median (interquartile range) or as No. (%). t-PA indicates tissue-type plasminogen activator; CHD, coronary heart disease; MI, myocardial infarction; TIA, transient ischemic attack; SBP, systolic blood pressure; DBP, diastolic blood pressure; mRS, modified Rankin Scale; NIHSS, National Institutes of Health Stroke Scale; TOAST, Trial of ORG 10172 in Acute Stroke Treatment; LAA, large artery atherosclerosis; LDL, low density lipoprotein.

**Supplementary Table 9. Comparison of 90-Day Outcomes in Patients with Aspirin and Intravenous t-PA in Symptomatic Large Vessel Occlusion Using Propensity Score Matching**

| Group | Outcome | Aspirin/t-PA  (%) | OR (95%CI) | *P* value |
| --- | --- | --- | --- | --- |
|  |  |  |  |  |
| All | mRS 0-1 | 73.15/88.07 | 0.37 (0.18, 0.76) | 0.01 |
|  | mRS 0-2 | 87.04/93.58 | 0.46 (0.18, 1.19) | 0.11 |
|  | Recurrent stroke | 10.91/8.18 | 1.37 (0.55, 3.41) | 0.49 |
|  | Recurrent ischemic stroke | 10.91/7.27 | 1.56 (0.61, 3.98) | 0.35 |
|  | All-cause mortality | 5.45/0.00 | - | - |
| Disabling | mRS 0-1 | 50.00/80.00 | 0.25 (0.06, 1.05) | 0.06 |
|  | mRS 0-2 | 85.71/88.00 | 0.82 (0.12, 5.59) | 0.84 |
|  | Recurrent stroke | 21.43/8.00 | 3.14 (0.46, 21.57) | 0.25 |
|  | Recurrent ischemic stroke | 21.43/8.00 | 3.14 (0.46, 21.57) | 0.25 |
|  | All-cause mortality | 0.00/0.00 | - | - |
| Non-disabling | mRS 0-1 | 76.60/90.48 | 0.34 (0.14, 0.82) | 0.02 |
|  | mRS 0-2 | 87.23/95.24 | 0.34 (0.11, 1.10) | 0.07 |
|  | Recurrent stroke | 9.38/8.24 | 1.15 (0.41, 3.24) | 0.79 |
|  | Recurrent ischemic stroke | 9.38/7.06 | 1.36 (0.46, 4.00) | 0.57 |
|  | All-cause mortality | 6.25/0.00 | - | - |
| Baseline NIHSS of 0-2 | mRS 0-1 | 78.85/89.58 | 0.43 (0.14, 1.36) | 0.15 |
|  | mRS 0-2 | 90.38/93.75 | 0.63 (0.14, 2.78) | 0.54 |
|  | Recurrent stroke | 3.77/8.33 | 0.43 (0.08, 2.47) | 0.34 |
|  | Recurrent ischemic stroke | 3.77/6.25 | 0.59 (0.09, 3.68) | 0.57 |
|  | All-cause mortality | 1.89/0.00 | - | - |
| Baseline NIHSS of 3-5 | mRS 0-1 | 67.86/86.89 | 0.32 (0.13, 0.81) | 0.02 |
|  | mRS 0-2 | 83.93/93.44 | 0.37 (0.11, 1.27) | 0.11 |
|  | Recurrent stroke | 17.54/8.06 | 2.43 (0.78, 7.59) | 0.13 |
|  | Recurrent ischemic stroke | 17.54/8.06 | 2.43 (0.78, 7.59) | 0.13 |
|  | All-cause mortality | 8.77/0.00 | - | - |

NIHSS indicates National Institutes of Health Stroke Scale; mRS modified Rankin Scale; t-PA, tissue-type plasminogen activator.

**Supplementary Table 10. Baseline Characteristics of Patients with DAPT and Intravenous t-PA in Symptomatic Large Vessel Occlusion Using Propensity Score Matching**

| Variables | Total | Intravenous  t-PA | DAPT | *P* value |
| --- | --- | --- | --- | --- |
|  | (N=254) | (N=127) | (N=127) |  |
| Age, y | 62.0 (55.0-69.0) | 62.0 (55.0-67.0) | 63.0 (56.0-71.0) | 0.28 |
| Male | 185 (72.8) | 94 (74.0) | 91 (71.7) | 0.67 |
| Current smoking | 88 (34.6) | 49 (38.6) | 39 (30.7) | 0.19 |
| Hypertension | 172 (67.7) | 90 (70.9) | 82 (64.6) | 0.28 |
| Diabetes mellitus | 48 (18.9) | 26 (20.5) | 22 (17.3) | 0.52 |
| Dyslipidemia | 23 (9.1) | 12 (9.4) | 11 (8.7) | 0.83 |
| Prior CHD/MI | 23 (9.1) | 13 (10.2) | 10 (7.9) | 0.51 |
| Atrial fibrillation | 8 (3.1) | 6 (4.7) | 2 (1.6) | 0.15 |
| Prior TIA | 8 (3.1) | 3 (2.4) | 5 (3.9) | 0.47 |
| Prior Stroke | 58 (22.8) | 28 (22.0) | 30 (23.6) | 0.77 |
| Random blood glucose, mmol/L | 6.8 (5.6-8.6) | 6.8 (5.6-15.6) | 7.0 (5.6-8.6) | 0.97 |
| Baseline SBP, mmHg | 147.3 (136.0-161.0) | 149.0 (139.0-165.0) | 143.5 (135.0-160.0) | 0.10 |
| Baseline DBP, mmHg | 86.0 (78.5-95.5) | 86.5 (78.5-95.5) | 85.0 (78.5-96.0) | 0.66 |
| Weight, kg | 70.0 (64.0-76.0) | 69.0 (62.0-77.0) | 70.0 (65.0-75.0) | 0.43 |
| Pre-mRS 0-1 | 238 (93.7) | 120 (94.5) | 118 (92.9) | 0.61 |
| Baseline NIHSS | 3.0 (1.0-4.0) | 3.0 (1.0-4.0) | 3.0 (1.0-4.0) | 0.86 |
| Care in stroke unit | 84 (33.1) | 48 (37.8) | 36 (28.3) | 0.11 |
| TOAST subtype |  |  |  | 0.79 |
| LAA | 214 (84.3) | 109 (85.8) | 105 (82.7) |  |
| Other determined cause | 9 (3.5) | 4 (3.1) | 5 (3.9) |  |
| Undetermined cause | 31 (12.2) | 14 (11.0) | 17 (13.4) |  |
| Medication history |  |  |  |  |
| Antiplatelet | 28 (11.0) | 11 (8.7) | 17 (13.4) | 0.23 |
| Lipid lowering agents | 20 (7.9) | 7 (5.5) | 13 (10.2) | 0.16 |
| Laboratory findings |  |  |  |  |
| Hemoglobin, g/L | 144.0 (134.0-153.0) | 144.0 (133.0-155.0) | 143.0 (134.0-151.0) | 0.37 |
| Platelet count, ×10^9^/L | 215.0 (177.0-258.0) | 216.0 (185.0-258.0) | 205.0 (173.0-260.0) | 0.18 |
| Serum creatinine, μmol/L | 69.3 (58.0-81.0) | 69.0 (57.0-78.0) | 70.0 (58.0-83.0) | 0.61 |
| LDL cholesterol, mmol/L | 2.4 (1.8-3.0) | 2.4 (1.8-3.0) | 2.4 (1.9-2.9) | 0.89 |

Values are presented as median (interquartile range) or as No. (%). DAPT indicates dual antiplatelet treatment; t-PA, tissue-type plasminogen activator; CHD, coronary heart disease; MI, myocardial infarction; TIA, transient ischemic attack; SBP, systolic blood pressure; DBP, diastolic blood pressure; mRS, modified Rankin Scale; NIHSS, National Institutes of Health Stroke Scale; TOAST, Trial of ORG 10172 in Acute Stroke Treatment; LAA, large artery atherosclerosis; LDL, low density lipoprotein.

**Supplementary Table 11. Comparison of 90-Day Outcomes in Patients with DAPT and Intravenous t-PA in Symptomatic Large Vessel Occlusion Using Propensity Score Matching**

| Group | Outcome | DAPT/t-PA  (%) | OR (95%CI) | *P* value |
| --- | --- | --- | --- | --- |
|  |  |  |  |  |
| All | mRS 0-1 | 80.16/84.92 | 0.72 (0.37, 1.38) | 0.32 |
|  | mRS 0-2 | 93.65/91.27 | 1.41 (0.55, 3.63) | 0.48 |
|  | Recurrent stroke | 9.45/10.24 | 0.92 (0.40, 2.09) | 0.83 |
|  | Recurrent ischemic stroke | 9.45/9.45 | 1.00 (0.43, 2.32) | 1.00 |
|  | All-cause mortality | 1.57/0.00 | - | - |
| Disabling | mRS 0-1 | 71.43/75.86 | 0.80 (0.22, 2.84) | 0.72 |
|  | mRS 0-2 | 85.71/82.76 | 1.25 (0.26, 5.93) | 0.78 |
|  | Recurrent stroke | 14.29/13.79 | 1.04 (0.21, 5.24) | 0.96 |
|  | Recurrent ischemic stroke | 14.29/13.79 | 1.04 (0.21, 5.24) | 0.96 |
|  | All-cause mortality | 0.00/0.00 | - | - |
| Non-disabling | mRS 0-1 | 81.90/87.63 | 0.64 (0.29, 1.40) | 0.26 |
|  | mRS 0-2 | 95.24/93.81 | 1.32 (0.39, 4.47) | 0.66 |
|  | Recurrent stroke | 8.49/9.18 | 0.92 (0.35, 2.41) | 0.86 |
|  | Recurrent ischemic stroke | 8.49/8.16 | 1.04 (0.39, 2.82) | 0.93 |
|  | All-cause mortality | 1.89/0.00 | - | - |
| Baseline NIHSS of 0-2 | mRS 0-1 | 83.93/89.29 | 0.63 (0.21, 1.90) | 0.41 |
|  | mRS 0-2 | 94.64/94.64 | 1.00 (0.19, 5.18) | 1.00 |
|  | Recurrent stroke | 8.93/8.93 | 1.00 (0.27, 3.67) | 1.00 |
|  | Recurrent ischemic stroke | 8.93/7.14 | 1.27 (0.32, 5.02) | 0.73 |
|  | All-cause mortality | 1.79/0.00 | - | - |
| Baseline NIHSS of 3-5 | mRS 0-1 | 77.14/81.43 | 0.77 (0.34, 1.75) | 0.53 |
|  | mRS 0-2 | 92.86/88.57 | 1.68 (0.52, 5.41) | 0.39 |
|  | Recurrent stroke | 9.86/11.27 | 0.86 (0.29, 2.52) | 0.79 |
|  | Recurrent ischemic stroke | 9.86/11.27 | 0.86 (0.29, 2.52) | 0.79 |
|  | All-cause mortality | 1.41/0.00 | - | - |

NIHSS indicates National Institutes of Health Stroke Scale; mRS modified Rankin Scale; t-PA, tissue-type plasminogen activator; DAPT, dual antiplatelet treatment.

**Supplementary Table 12. Baseline Characteristics of Patients in Asymptomatic Large Vessel Occlusion**

| Variables | Total | Intravenous  t-PA | DAPT | Aspirin | *P* value |
| --- | --- | --- | --- | --- | --- |
|  | (N=639) | (N=112) | (N=320) | (N=207) |  |
| Age, y | 63.0 (55.0-70.0) | 62.0 (55.0-69.0) | 63.0 (55.0-68.5) | 64.0 (56.0-73.0) | 0.07 |
| Male | 451 (70.6) | 77 (68.8) | 227 (70.9) | 147 (71.0) | 0.90 |
| Current smoking | 221 (34.6) | 38 (33.9) | 119 (37.2) | 64 (30.9) | 0.33 |
| Hypertension | 403 (63.1) | 71 (63.4) | 205 (64.1) | 127 (61.4) | 0.82 |
| Diabetes mellitus | 167 (26.1) | 24 (21.4) | 96 (30.0) | 47 (22.7) | 0.08 |
| Dyslipidemia | 48 (7.5) | 8 (7.1) | 25 (7.8) | 15 (7.2) | 0.96 |
| Prior CHD/MI | 61 (9.5) | 6 (5.4) | 32 (10.0) | 23 (11.1) | 0.23 |
| Atrial fibrillation | 21 (3.3) | 8 (7.1) | 5 (1.6) | 8 (3.9) | 0.01 |
| Prior TIA | 11 (1.7) | 0 (0) | 4 (1.3) | 7 (3.4) | 0.06 |
| Prior Stroke | 129 (20.2) | 16 (14.3) | 69 (21.6) | 44 (21.3) | 0.23 |
| Random blood glucose, mmol/L | 7.2 (6.1-9.7) | 5.8 (5.6-7.0) | 7.2 (6.1-12.9) | 7.9 (6.8-11.0) | 0.03 |
| Baseline SBP, mmHg | 150.0 (138.0-165.5) | 154.8 (142.8-170.0) | 150.0 (137.5-163.5) | 150.0 (138.0-164.0) | 0.07 |
| Baseline DBP, mmHg | 85.5 (79.5-95.0) | 89.0 (80.0-98.3) | 85.0 (80.0-95.0) | 85.0 (79.0-94.5) | 0.21 |
| Weight, kg | 70.0 (61.0-75.0) | 68.5 (60.0-75.0) | 70.0 (61.0-75.5) | 70.0 (62.5-75.0) | 0.37 |
| Pre-mRS 0-1 | 592 (92.6) | 105 (93.8) | 294 (91.9) | 193 (93.2) | 0.75 |
| Baseline NIHSS | 2.0 (1.0-4.0) | 3.0 (2.0-4.0) | 2.0 (1.0-4.0) | 2.0 (1.0-4.0) | 0.01 |
| Care in stroke unit | 148 (23.2) | 51 (45.5) | 62 (19.4) | 35 (16.9) | <0.001 |
| TOAST subtype |  |  |  |  | 0.06 |
| Cardioembolic | 57 (8.9) | 17 (15.2) | 21 (6.6) | 19 (9.2) |  |
| SAO | 239 (37.4) | 41 (36.6) | 120 (37.5) | 78 (37.7) |  |
| Other determined cause | 13 (2.0) | 2 (1.8) | 10 (3.1) | 1 (0.5) |  |
| Undetermined cause | 330 (51.6) | 52 (46.4) | 169 (52.8) | 109 (52.7) |  |
| Medication history |  |  |  |  |  |
| Antiplatelet | 101 (15.8) | 12 (10.7) | 50 (15.6) | 39 (18.8) | 0.16 |
| Anticoagulant | 6 (0.9) | 2 (1.8) | 1 (0.3) | 3 (1.4) | 0.25 |
| Lipid lowering agents | 64 (10.0) | 7 (6.3) | 30 (9.4) | 27 (13.0) | 0.13 |
| Laboratory findings |  |  |  |  |  |
| Hemoglobin, g/L | 142.0 (133.0-151.0) | 143.0 (135.0-150.0) | 142.0 (133.0-151.0) | 141.0 (131.0-152.0) | 0.84 |
| Platelet count, ×10^9^/L | 206.5 (175.0-243.0) | 211.5 (180.0-244.0) | 214.0 (178.0-245.0) | 196.0 (168.0-241.0) | 0.07 |
| Serum creatinine, μmol/L | 71.0 (61.0-81.4) | 69.3 (62.0-79.0) | 70.0 (59.0-82.0) | 72.0 (63.0-83.0) | 0.26 |
| LDL cholesterol, mmol/L | 2.5 (1.9-3.0) | 2.4 (1.7-2.9) | 2.5 (1.9-3.2) | 2.4 (1.8-3.0) | 0.22 |

Values are presented as median (interquartile range) or as No. (%). DAPT indicates dual antiplatelet treatment; t-PA, tissue-type plasminogen activator; CHD, coronary heart disease; MI, myocardial infarction; TIA, transient ischemic attack; SBP, systolic blood pressure; DBP, diastolic blood pressure; mRS, modified Rankin Scale; NIHSS, National Institutes of Health Stroke Scale; TOAST, Trial of ORG 10172 in Acute Stroke Treatment; SAO, small artery occlusion; LDL, low density lipoprotein.

**Supplementary Table 13. Main Outcomes in Asymptomatic Large Vessel Occlusion**

| Outcome | Group | Event/N (%) | Unadjusted | |  | Multivariable adjusted* | |
| --- | --- | --- | --- | --- | --- | --- | --- |
|  |  |  | OR (95%CI) | *P* value |  | OR (95%CI) | *P* value |
| mRS 0-1 at 90 days | Intravenous t-PA | 99/112 (88.39) | Ref. |  |  | Ref. |  |
|  | DAPT | 272/316 (86.08) | 0.81 (0.42, 1.57) | 0.54 |  | 0.72 (0.35, 1.47) | 0.36 |
|  | Aspirin | 160/202 (79.21) | 0.50 (0.26, 0.98) | 0.04 |  | 0.47 (0.22, 0.98) | 0.045 |
| mRS 0-2 at 90 days | Intravenous t-PA | 110/112 (98.21) | Ref. |  |  | Ref. |  |
|  | DAPT | 303/316 (95.89) | 0.42 (0.09, 1.91) | 0.26 |  | 0.48 (0.10, 2.26) | 0.35 |
|  | Aspirin | 188/202 (93.07) | 0.24 (0.05, 1.09) | 0.07 |  | 0.35 (0.07, 1.66) | 0.19 |
| Recurrent stroke at 90 days | Intravenous t-PA | 6/112 (5.36) | Ref. |  |  | Ref. |  |
|  | DAPT | 9/320 (2.81) | 0.51 (0.18, 1.47) | 0.21 |  | 0.56 (0.18, 1.71) | 0.31 |
|  | Aspirin | 10/207 (4.83) | 0.90 (0.32, 2.54) | 0.84 |  | 1.04 (0.33, 3.23) | 0.95 |
| Recurrent ischemic stroke at 90 days | Intravenous t-PA | 6/112 (5.36) | Ref. |  |  | Ref. |  |
|  | DAPT | 9/320 (2.81) | 0.51 (0.18, 1.47) | 0.21 |  | 0.56 (0.18, 1.71) | 0.31 |
|  | Aspirin | 10/207 (4.83) | 0.90 (0.32, 2.54) | 0.84 |  | 1.04 (0.33, 3.23) | 0.95 |
| All-cause mortality at 90 days | Intravenous t-PA | 0/112 (0) | Ref. |  |  | Ref. |  |
|  | DAPT | 1/320 (0.31) | - | - |  | - | - |
|  | Aspirin | 2/207 (0.97) | - | - |  | - | - |

*Adjusted for age, sex, current smoking, diabetes mellitus, atrial fibrillation, prior TIA, random blood glucose, baseline SBP, pre-mRS, baseline NIHSS, care in stroke unit, TOAST subtype, medication history of antiplatelet and lipid-lowering agents, platelet count, and serum creatinine.

TIA indicates transient ischemic attack; SBP, systolic blood pressure; mRS, modified Rankin Scale; NIHSS, National Institutes of Health Stroke Scale; DAPT, dual antiplatelet treatment; t-PA, tissue-type plasminogen activator.


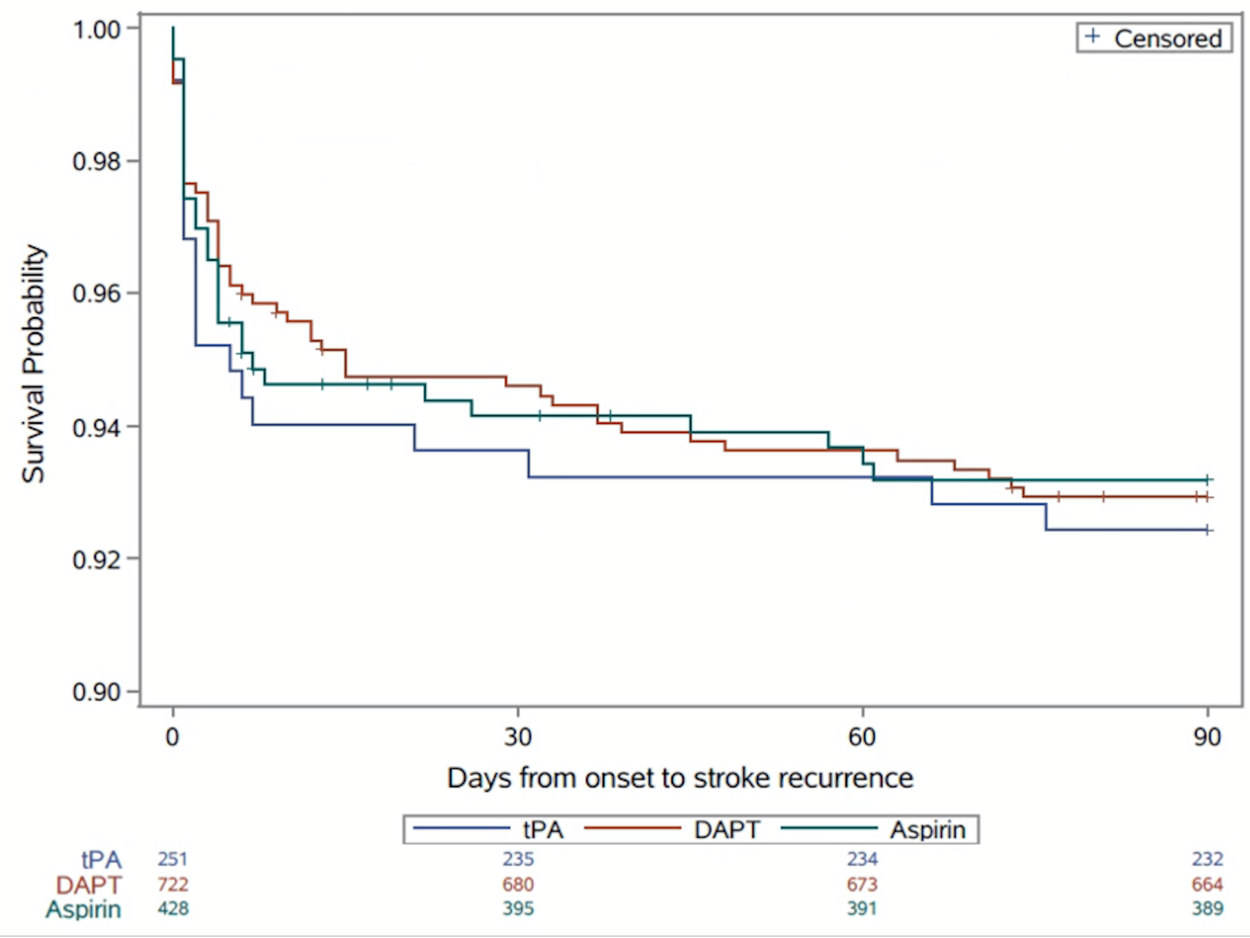


**Supplementary Figure 1. Kaplan-Meier Curve for the Recurrent Stroke at 90 Days**


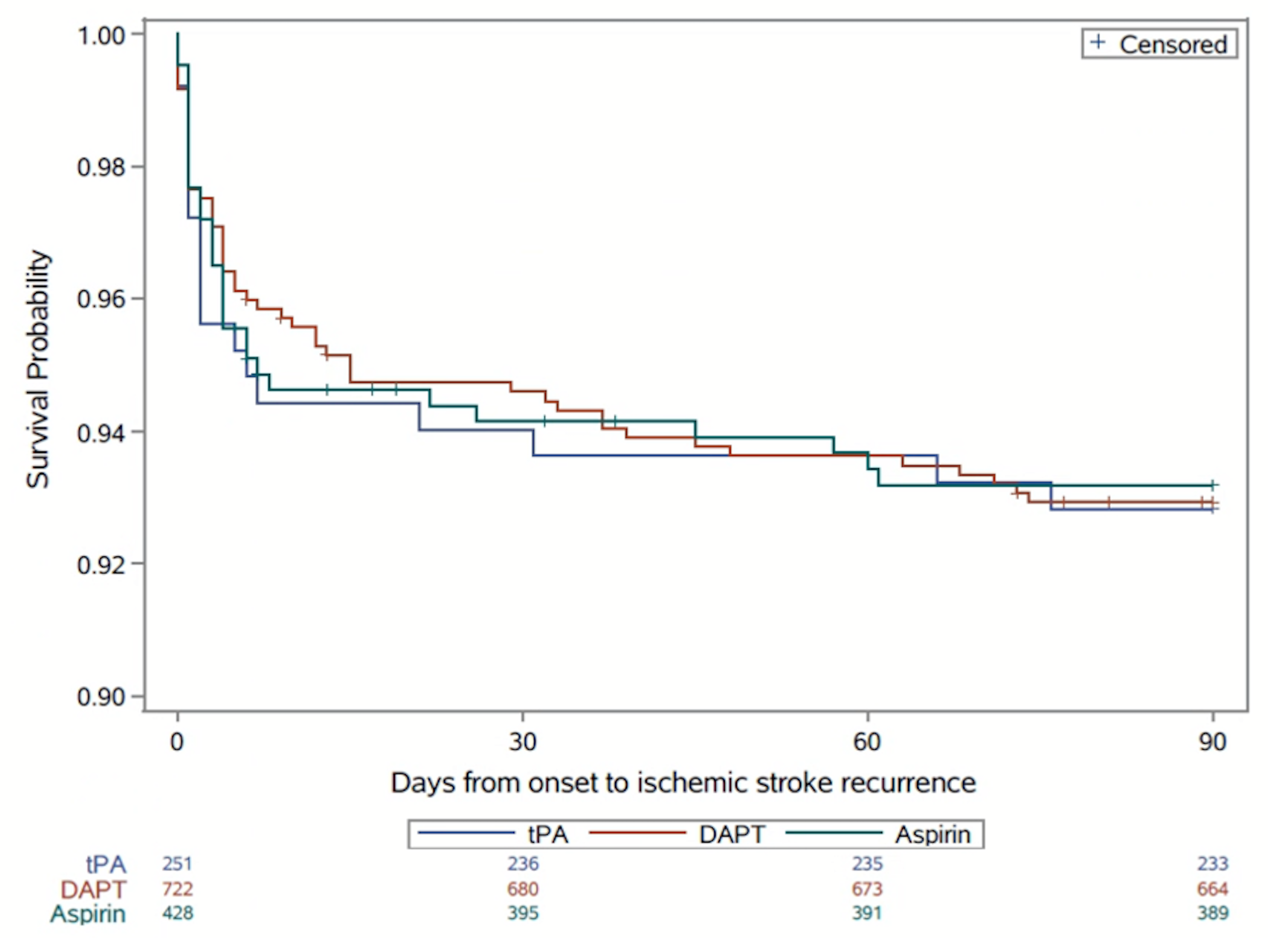


**Supplementary Figure 2. Kaplan-Meier Curve for the Recurrent Ischemic Stroke at 90 Days**
